# Longitudinal study of visual field changes determined by Humphrey Field Analyzer 10-2 in patients with Retinitis Pigmentosa

**DOI:** 10.1038/s41598-017-16640-7

**Published:** 2017-11-27

**Authors:** Akira Sayo, Shinji Ueno, Taro Kominami, Kazuki Nishida, Daiki Inooka, Ayami Nakanishi, Shunsuke Yasuda, Satoshi Okado, Kunihiko Takahashi, Shigeyuki Matsui, Hiroko Terasaki

**Affiliations:** 1 Department of Ophthalmology, Nagoya University Graduate School of Medicine, Nagoya, Japan; 2 Department of Biostatistics, Nagoya University Graduate School of Medicine, Nagoya, Japan

## Abstract

The aim of this study is to determine the progress of the visual field defects obtained by the Humphrey Field Analyzer 10-2 program (HFA 10-2) in patients with retinitis pigmentosa (RP). The medical records of 45 eyes of 45 RP patients who had at least 3 visual field tests were reviewed. Linear mixed models were used to follow the changes of the mean deviation and the average sensitivity of 4, 12, and 20 points in three concentric squares, designated as S4, S12, and S20. The median follow-up time was 3.86 years [range: 1.93 to 9.86, IQR (Interquartile range): 3.01 to 4.93]. The median number of the visual field tests was 3 (range: 3 to 15, IQR: 3 to 4). The mean change of the MD was −0.46 dB/year (−5.80%/year). When the patients were grouped by the average initial MD, the less advanced group had slower progressions than the more advanced group in S4, S12, and S20. These results should be useful in understanding the pathological changes of RP in the central visual field.

## Introduction

Retinitis pigmentosa (RP) is a genetically heterogeneous group of inherited retinal disorders characterized by a slow, progressive loss of photoreceptors primarily the rods. The characteristic fundus changes include that of the retinal pigment epithelium (RPE), blood vessel attenuation, disc pallor, and presence of bone-spicule pigmentation, which can be seen at different stages of RP. The signs and symptoms of RP are impaired night vision and slow progressive visual field loss and eventual decline in the visual acuity. The loss of the cone photoreceptors results in the central visual field loss and visual acuity reduction, which are the most critical problems for RP patients. Visual field loss progresses peripheral-to-central but can simultaneously expand peripherally and centrally in patients with ring scotomas.

Several therapeutic options are present for the preclinical or early clinical stages of RP, and the methods used to assess the efficacy of these treatments are being extensively studied. The natural history of RP must be known to determine the possible clinical markers of a progression of RP, and these can be used to evaluate the safety and efficacy of new treatments.

The time course of the progression of the visual field defects in RP has been extensively studied by Goldmann kinetic perimetry^1–4^, and the results have shown several patterns of visual field progression; the basic pattern of concentric visual field loss, changes beginning with scotomas in the mid-peripheral regions, asymmetrical visual field loss, and the end stage when only the central visual field remains^5,6^. These findings indicated that an evaluation of the central visual field is essential in the advanced stage of RP, however Goldmann kinetic perimetry is not appropriate for evaluating small areas of the central visual field^3,5^.

Automated static perimetry with the Humphrey Field Analyzer (HFA; Carl Zeiss Meditec, Inc. Dublin, CA, USA) is a reliable method to evaluate the central visual filed, and it is widely used to determine the stage of glaucoma. The HFA has been used to assess not only the extent of the central visual field but also to quantify the visual sensitivity of different areas of the central visual field. Two studies have shown a progressive constriction of the visual field in RP patients with the HFA 30-2 program^7,8^. Several other studies have demonstrated the usefulness of the HFA 10-2 program to evaluate RP patients^9–12^. Because RP patients have difficulties when the visual field constricts to the central 10 degrees, the HFA 10-2 program is a reasonable option for assessing the disease progression. However, earlier studies included only a small number of RP patients^10,11^, and the nature of progression of the visual field in the central 10 degrees was not determined conclusively.

Thus, the aim of this study was to assess the annual decrease of the visual sensitivity of the central visual field in RP patients. We reviewed the serial results of the sensitivities determined by the HFA 10-2 program in RP patients and performed trend analysis with mixed linear models. We determined the annual progression of visual field sensitivity decrease of the mean deviation (MD) and also the sensitivities of three concentric areas of the HFA 10-2 by a method that was a modification of previous studies^11,13^.

## Results

### Clinical data

The demographic and clinical characteristics of the 45 patients (22 men, 23 women) with RP are shown in Table 1. The median age at the baseline was 47.2 years (range: 14.1 to 70.2, IQR: 37.5 to 58.1). Of the 45 patients, 9 had autosomal dominant RP, 11 had autosomal recessive RP, 1 had X-linked RP, and 24 were diagnosed as simplex RP. The type of the inheritance was based mainly on the family history, and genetic testing was conducted on 6 cases. The median follow-up period was 3.86 years (range: 1.93 to 9.86, IQR: 3.01 to 4.93). The median number of the visual field tests was 3 (range: 3 to 15, IQR: 3 to 4)

**Table 1:** Summary of 45 patients’ clinical data.

Men	22
Women	23
Autosomal dominant	9
Autosomal recessive	11
X-linked	1
Simplex	24
Median baseline MD (dB)	−18.8
Range	[−28.3 to −6.17]
IQR	[−22.5 to −13.6]
Median baseline S4 (dB)	−4.64
Range	[−21.7 to 0.27]
IQR	[−8.31 to −3.00]
Median baseline S12 (dB)	−8.68
Range	[−23.6 to 0.59]
IQR	[−13.7 to −5.01]
Median baseline S20 (dB)	−14.6
Range	[−29.6 to −1.09]
IQR	[−16.5 to −10.4]
Median baseline visual acuity (logMAR)	0.8
Range	[0.09 to 1.30]
IQR	[0.50 to 1.00]
Median baseline age (years)	47.2
Range	[14.1 to 70.2]
IQR	[37.5 to 58.1]
Median follow-up time (years)	3.86
Range	[1.93 to 9.86]
IQR	[3.01 to 4.93]
Median number of HFA10-2	3
Range	[3 to 15]
IQR	[3 to 4]

IQR = Interquartile range.

MAR = Minimum angle of resolution.

**Figure 1:**
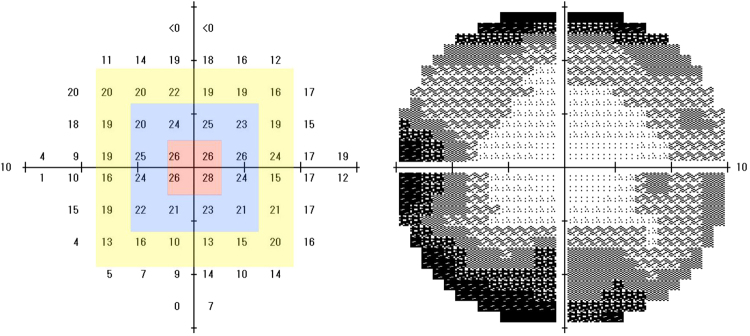
Representative visual fields of a patient with retinitis pigmentosa (RP). The central retina is divided into three concentric squares. The overall visual field size is 10 degrees. The numerical display (left) and grey scale display (right) of the HFA 10-2 are shown. The average sensitivity of the 4 central points (red), the surrounding 12 (blue), and 20 points (yellow) are designated as the S4, S12, and S20 areas respectively.

The median visual sensitivities of the HFA 10-2 are also shown in Table 1, and the division of the HFA 10-2 into three concentric squares is shown in Fig. 1. The average and median initial MD of the 45 patients at the baseline were −17.9 dB and −18.8 dB (range: −28.3 to −6.17, IQR: −22.5 to −13.6), respectively. The median sensitivities were −4.64 dB (range: −21.7 to 0.27, IQR: −8.31 to −3.00) for S4, −8.68 dB (range: −23.6 to 0.59, IQR: −13.7 to −5.01) for S12, and −14.6 dB (range: −29.6 to −1.09, IQR: −16.5 to −10.4) for S20.

### Rate of progression

**Figure 2:**
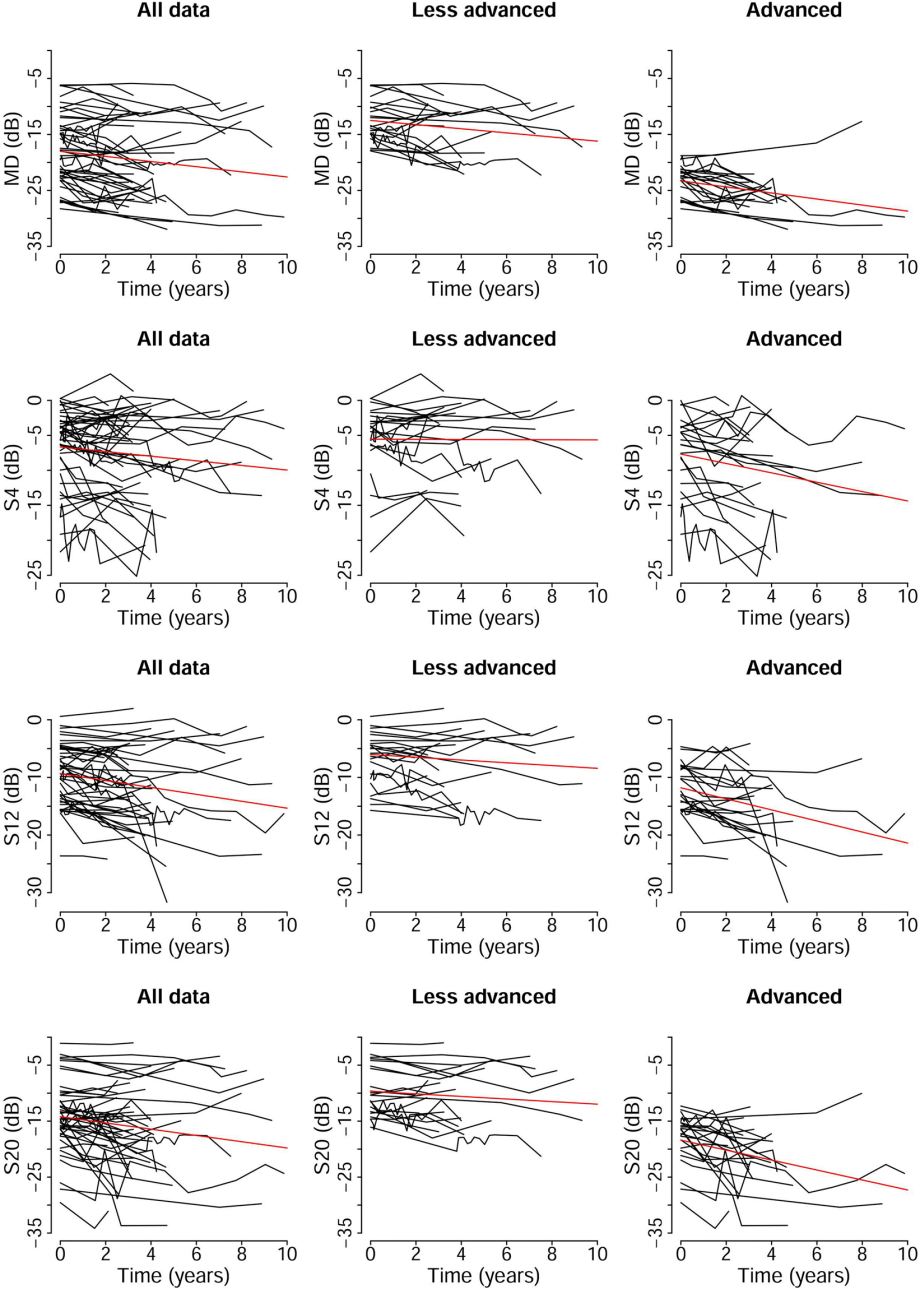
Plots of the mean deviations (MD) and the visual sensitivities as a function of the follow-up years. The mean MDs of all sections are shown in the top row, the means of S4 in the second row, the means of S12 in the third row, and the means of S20 in the fourth row. Data of 45 patients are shown in the left column. The data of patients whose initial MD was better than average (−17.9 dB) are shown in the middle column (n = 22), and the data of patients whose initial MD was worse than average are shown in the right column (n = 23). A red line is the best-fit regression line of each group.

The MD and sensitivity for S4, S12, and S20 at each follow-up time are plotted for each patient in Fig. 2. The calculated mean reduction of the MD was −0.46 dB/year [95% confidence interval (CI), −0.64 to −0.28; *P* < 0.001] or −5.80% (95% CI, −10.6 to −0.80; *P* = 0.023) on a linear scale (1/Lambert). Because dB is a logarithmic scale, we converted dB to 1/Lambert to calculate the yearly deterioration rate in percentages. The progression rate of sensitivity was −0.33 dB/year (95% CI, −0.59 to −0.05; *P* = 0.023) for S4, −0.60 dB/year (95% CI, −0.89 to −0.31; *P* < 0.001) for S12, and −0.56 dB/year (95% CI, −0.84 to −0.29; *P* < 0.001) for S20 (Table 2).

**Table 2:** Average progression rate of MD, S4, S12, and S20.

Outcome	Coefficient	95% CI	*P* value
MD			
Intercept	−18.0	−19.9 to −16.1	<0.001
Time (years)	−0.46	−0.64 to −0.28	<0.001
S4			
Intercept	−6.66	−8.24 to −5.07	<0.001
Time (years)	−0.33	−0.59 to −0.05	0.023
S12			
Intercept	−9.34	–10.9 to −7.78	<0.001
Time (years)	−0.60	–0.89 to −0.31	<0.001
S20			
Intercept	−14.2	−16.0 to −12.3	<0.001
Time (years)	−0.56	−0.84 to −0.29	<0.001

Summary of 4 linear mixed models. For each, the outcome is MD, S4, S12, and S20. All models include random intercepts and random slopes for each subject over time. Coefficient of time (years) of each model is the fixed effect of the slopes, or the average progression rate. Units of the outcome are in dB. CI = Confidence Interval.

### Interactions between visual sensitivities and other factors

Linear mixed models were fit to the interaction terms to determine whether the changes in the MD were affected by other factors. We examined possible interactions with age, sex, mode of inheritance (autosomal dominant or not), and the baseline MD. In terms of baseline MD, patients were divided into those with the initial MD of ≥−17.9 dB, which was the average initial MD of the patients, (less advanced group) and <−17.9 dB (advanced group). None of the above factors had statistically significant interaction between times on MD progression rate (Table 3).

**Table 3:** Analysis of the effect of variables on progression rate.

Outcome	Coefficient	95% CI	*P* value
MD			
Intercept	−23.3	−24.7 to −21.9	<0.001
Time (years)	−0.54	−0.80 to −0.28	<0.001
≥−17.9 dB	10.8	8.81 to 12.8	<0.001
Time *≥−17.9 dB	0.17	−0.19 to 0.54	0.366
MD			
Intercept	−13.2	−19.2 to −7.12	<0.001
Time (years)	0.17	−0.48 to 0.81	0.614
Age	−0.11	−0.23 to 0.02	0.101
Time * Age	−0.01	−0.02 to 0.002	0.102
MD			
Intercept	−15.3	−17.6 to −12.9	<0.001
Time (years)	−0.35	−0.61 to −0.10	0.009
Men	−5.68	−9.07 to −2.29	0.002
Time * Men	−0.20	−0.56 to 0.15	0.276
MD			
Intercept	−17.7	−19.8 to −15.6	<0.001
Time (years)	−0.45	−0.66 to −0.25	<0.001
AD	−1.51	−6.22 to 3.20	0.535
Time * AD	−0.01	−0.47 to 0.44	0.955

Summary of 4 linear mixed models. For each, the outcome is MD. All models include one of the variables (baseline MD, Age, Sex, and AD), time (years), and an interaction between the variable and time, as well as random intercepts and slopes for time for each subject. The patients were divided into two groups by the average initial MD (−17.9 dB). Units of the outcome are in dB.

AD = Autosomal Dominant, CI = Confidence Interval.

We also tested whether the baseline MD would affect the progression rate in S4, S12, and S20. The visual sensitivities of the advanced group had a faster decrease in all of the concentric square areas (Table 4). More specifically, the rate of progression of S4, S12, and S20 were −0.67 dB/year, −0.96 dB/year, −0.89 dB/year in the advanced group, and −0.01 dB/year, −0.24 dB/year, −0.23 dB/year in the less advanced group (*P* = 0.016, *P* = 0.013, *P* < 0.001, respectively).

**Table 4:** Analysis of the effect of baseline MD on progression rate in S4, S12, and S20.

Outcome	Coefficient	95% CI	*P* value
S4			
Intercept	−7.68	−9.86 to −5.50	<0.001
Time (years)	−0.67	−1.04 to −0.30	<0.001
≥−17.9 dB	2.13	−0.98 to 5.26	0.189
Time *≥−17.9 dB	0.66	0.15 to 1.19	0.016
S12			
Intercept	−11.8	−13.7 to −9.90	<0.001
Time (years)	−0.96	−1.35 to −0.57	<0.001
≥−17.9 dB	5.06	2.33 to 7.79	<0.001
Time *≥−17.9 dB	0.72	0.18 to 1.26	0.013
S20			
Intercept	−18.4	−20.2 to −16.6	<0.001
Time (years)	−0.89	−1.26 to −0.51	<0.001
≥−17.9 dB	8.73	6.07 to 11.4	<0.001
Time *≥−17.9 dB	0.66	0.14 to 1.19	0.016

Summary of 3 linear mixed models. For each, the outcome is S4, S12, and S20. All models include baseline MD, time (years), and an interaction between baseline MD and time, as well as random intercepts and slopes for time for each subject. The patients were divided into two groups by the average initial MD (−17.9 dB). Units of the outcome are in dB. CI = Confidence Interval.

## Discussion

Our results indicated that the average MD progression rate of 45 patients was −0.46 dB/year, (95% CI −0.64 to −0.28) with a *P* value < 0.001. This value was significantly lower than 0, which indicates that the reduction of the visual field was a real progression. Although several studies have shown a decrease of the visual field sensitivities obtained by HFA10-2 in RP patients, only a few studies reported the rate of the natural progression of the visual sensitivities using the regression coefficient of the linear MD slopes^10,11,14^. A small, non-masked prospective study by Nakazawa *et al*. showed that oral calcium-channel blocker, nilvadipine, led to a slower progression of the visual field deterioration in the treated RP patients than in the non-treated control RP group^10,14^. In these studies, the mean MD slope for total observational periods of 49.2 months of 14 control RP patients was −0.89 dB/year and that of 65.7 months in 22 control RP patients was −1.37 dB/year for the right eye and −1.15 dB/year for the left eyes. They also analyzed the central four points of the HFA10-2 (CENT4) visual field, i.e., S4. The average regression coefficient was −1.29 dB/year for the right eyes and −1.75 dB/year for the left eyes in the control group. The visual sensitivity deterioration rates in the control group of Nakazawa’s studies were faster than our rates in RP patients. The reasons for the difference in the progression rates might be related to the study design, prospective vs retrospective, and/or the difference of stage of the studied RP patients. The mean sensitivity for S4 of our RP patients was better than that of their control RP patients^14^, which indicated that the more central visual fields were more impaired in their patients. Of note, CENT4 in their study was calculated without conversion of dB to 1/Lambert, which could have made the difference. We also determined whether the baseline MD would affect the deterioration rate of sensitivity in S4, and the results showed that the less advanced group had a much slower progression than the advanced group (−0.01 dB/year vs −0.67 dB/year; *P* = 0.016). The results were reasonable because it was known that the central visual field remained preserved until the advanced stage of RP. The faster reduction in S12 and S20 in the advanced group might be due to the same reason.

The natural history of the visual field changes of RP eyes has been extensively assessed by Goldmann perimetry. The rate of visual field loss as determined by Goldmann perimetry has been reported to be affected by differences of the stimulus target, inheritance pattern, and phenotype as well as other factors^1,3,6,15,16^. The rate of visual field loss ranged widely from 2.7%/year^17^ to 14.5%/year^18^ although many of the results were 5 to 8%/year^16,19^. Thus, our results are consistent with these Goldmann perimetric studies. However, we have to take into account that some of our patients might have lost visual sensitivities outside the central 10 degrees where our study evaluated.

Recent advancements of optical coherence tomography (OCT) have enabled clinicians to follow the microstructural changes in RP patients, and the low test-retest variability has made it possible to detect changes in a shorter time^20,21^. Several studies have reported a significant correlation between the presence of the ellipsoid zone (EZ) in the OCT images and the visual field sensitivities^22,23^. The visual sensitivities determined by HFA have been reported to be correlated with the EZ width^11^. Sujirakul *et al*. reported that the mean annual progression rates of the EZ in RP was 4.9%/year^20^. They also reported a significantly lower mean rate of microstructural disruption in patients with shorter EZ (<3000 mm) than longer EZ (>3000 mm). This indicated that the rate of disruption decreased as the disease approached the fovea^20^. An EZ length of 3000 mm corresponds to about 10 degrees in normal size eyes which corresponds to a visual field of 5 degrees. Their data are not consistent with our results, which had a steeper, although not significant, MD slope in patients with advanced baseline MD. Possible reasons for the discrepancy are: the visual impairment rate of the less advanced group was underestimated in our study because these patients might have lost visual sensitivity outside the 10 degrees of the central visual field, and the HFA10-2 might have detected the visual sensitivity loss where the EZ band was present. In addition, MD (dB) is measured on a log scale while the EZ on a linear scale. Therefore, the progression of the MD may have been underestimated in RP eyes at earlier stages^24^. Further analyses on the relationship between the HF10-2 determined sensitivity and the microstructures in the OCT image will be needed.

Some researchers have reported that RP patients with different inheritance patterns have different rates of progression of the visual sensitivities, but we could not confirm this because of the relatively small sample size^20,25^.

The difference in the rate of visual field decrease in RP eyes with different genotypes should be studied in the future. Our results showed that the age and sex did not affect the MD progression rate which is consistent with an earlier study^20^.

There are several limitations in our study. One is the small sample size, and another is the retrospective design. In addition, the visual field tests are subjective and have high test-retest variability. The results of some of our patients might have been affected by the learning effect. More frequent visual field analysis at shorter intervals should determine the course of the central visual field changes better. Fourth, we converted dB to 1/Lambert only when calculating the average sensitivity of concentric square areas and the yearly progression rate of MD in percentage. Our results could be totally different if we used this conversion in every analysis. However, clinicians are much more familiar with dB than 1/Lambert, and thus we employed this conversion only when necessary.

In conclusion, our results showed that the rate of decrease of the visual sensitivities of the central retina can be followed by HFA10-2. We conclude that this method is useful in determining the rate of progress of the disease process in RP patients and the results can be used in counseling patients.

## Methods

### Subjects

All of the procedures conformed to the tenets of the Declaration of Helsinki, and Institutional Review Board (IRB)/Ethics Committee approval was obtained (approval No. 2016-0538). The review board waived the need for written informed consent, because the study design comprised a retrospective chart review. We reviewed the medical records of 282 RP patients who were examined at the Nagoya University Hospital. The diagnosis of RP was based on; the presence of night blindness, typical fundus appearance including changes of the retinal pigment epithelium (RPE), blood vessel attenuation, bone spicule pigmentation, peripheral visual field loss, and a reduced (<50 µV) or extinguished full-field scotopic flash electroretinograms (ERGs; PE-300, TOMEY, Nagoya, Japan). From the 282 patients, 45 eyes of 45 patients were selected because they had had at least 3 visual field determinations by the HFA 10-2 at different times. The time points when we evaluated the visual field are shown in Fig. 2. Because of the retrospective design, we were not able to define the time points of visual field tests. We usually examine RP patients every 6 months to 1 year, so the intervals between HFA 10-2 were usually more than 6 months in most of the patients. However, when patients showed a rapid decrease in the visual field sensitivity or they were concerned about their condition, we performed the test within 6 months in a small number of cases. Eyes with other disorders severe enough to affect the visual field such as cystoid macular edema, epiretinal membrane, and vitreo-macular traction syndrome were excluded. The first and the second authors (AS and SU) independently excluded the patients, and when there was a disagreement, the third author (TK) made the final decision. Eyes whose baseline MD was less than −30 dB or more than −5.0 dB were excluded because our aim was to evaluate the patients whose visual field was impaired within the 10 degrees of the central visual field but with some visual field still preserved. Patients with HFA10-2 fixation loss scores more than 20% or false-positive or false-negative errors more than 33% were also excluded. If both eyes of an individual were eligible, the eye with the better MD at the baseline was chosen for the analysis.

### Division of visual fields of HFA 10-2

To characterize the visual field changes in more detail, we selected 3 concentric square areas centered on the fovea of the HFA 10-2 visual fields. One square contained 4 points, the next size square contained 12 points, and the third square contained 20 points. The squares were designated as S4, S12, and S20 (Fig. 1), and the overall size corresponded to visual fields of about 1, 3, and 5 degrees. When calculating the average sensitivities of S4, S12, and S20, we first converted dB (logarithmic scale) to 1/Lambert (linear scale), then averaged the values, and then converted the values back to dB^26^. We used the following equation for this conversion^24^.

$${\rm{MD}}\,(1/{\rm{Lambert}})={10}^{{\rm{MD}}({\rm{dB}})/10}$$

### Statistical analyses

The overall progress of the retinal sensitivities was fit by a linear trend analysis because the follow-up time was relatively short with small number of measurement points. Linear mixed models were used to determine the changes of the MD and sensitivity of S4, S12, and S20 with increasing time. Table 2 summarizes 4 models that assessed the average progression rates. For each model, the outcome was MD, S4, S12, and S20. All models included random intercepts and random slopes for each subject over time. Coefficient of time (years) of each model was the fixed effect of the slopes, or the average progression rate. To identify the factors that could affect the MD progression rate, interactions between time and the factors were tested. The factors examined were age, sex, mode of inheritance, and the baseline MD. Because the mode of inheritance was determined mainly from the family history, a clear distinction between simplex and autosomal recessive RP may not have been made. In addition, there was only one X-linked RP patient whose inheritance pattern was confirmed by genetic testing. Therefore, we only compared the progression rate between the autosomal dominant (AD) RP against the rest of the patients. To examine the effects of the baseline MD, we divided the patients into those whose MD at the initial field test was ≥−17.9 dB, which was the mean initial MD of 45 patients, and those whose initial MD was <−17.9 dB. Table 3 summarizes 4 models that analyzed the effect of the factors on MD progression rate. For each model, the outcome was MD. All models included one of the factors (baseline MD, age, sex, and AD), time (years), and an interaction between the factor and time, as well as random intercepts and slopes for time for each subject. Furthermore, to identify whether baseline MD would affect the progression rate in S4, S12, and S20, we constructed 3 mixed linear models (Table 4). For each, the outcome was S4, S12, and S20. All models included baseline MD, time (years), and an interaction between baseline MD and time, as well as random intercepts and slopes for time for each subject.

To determine the percentage MD reduction rate relative to the preserved visual field, we converted the MD values on a logarithmic scale to a linear scale using the equation described above. We then constructed a linear mixed model with MD (1/Lambert) as an outcome, which included random intercepts and random slopes for each subject over time. The yearly progression rate in percentage is represented as a decrease from the average initial MD (1/Lambert) of the cohort. All statistical analyses were performed with R version 3.2.3.
